# Butter naturally enriched in *cis*-9, *trans*-11 CLA prevents hyperinsulinemia and increases both serum HDL cholesterol and triacylglycerol levels in rats

**DOI:** 10.1186/1476-511X-13-200

**Published:** 2014-12-22

**Authors:** Mariana Macedo de Almeida, Sheila Cristina Potente Dutra Luquetti, Céphora Maria Sabarense, José Otávio do Amaral Corrêa, Larissa Gomes dos Reis, Ellen Paula Santos da Conceição, Patrícia Cristina Lisboa, Egberto Gaspar de Moura, Jacy Gameiro, Marco Antônio Sundfeld da Gama, Fernando César Ferraz Lopes, Raúl Marcel González Garcia

**Affiliations:** 1 Department of Biology, Federal University of Juiz de Fora, Juiz de Fora, Minas Gerais Brazil; 2 Department of Nutrition, Federal University of Juiz de Fora, Juiz de Fora, Minas Gerais Brazil; 3 Department of Pharmaceutical Sciences, Federal University of Juiz de Fora, Juiz de Fora, Minas Gerais Brazil; 4 Department of Physiological Sciences, Roberto Alcantara Gomes Institute of Biology, State University of Rio de Janeiro, Rio de Janeiro, Brazil; 5 Department of Parasitology, Microbiology and Immunology, Federal University of Juiz de Fora, Juiz de Fora, Minas Gerais Brazil; 6 Embrapa Dairy Cattle, Juiz de Fora, Juiz de Fora, Minas Gerais Brazil

**Keywords:** High conjugated linoleic acid enriched butter, Functional food, Rats, Insulin sensitivity, Dyslipidemia, Diabetes

## Abstract

**Background:**

Evidence from *in vitro* and animal studies indicates that conjugated linoleic acid (CLA) possesses anti-diabetic properties, which appear to be attributed to *cis*-9, *trans*-11 CLA, the major CLA isomer in ruminant fat. However, there is a shortage of studies addressing CLA from natural source. The present study aimed to evaluate the effects of butter naturally enriched in *cis*-9, *trans*-11 CLA on parameters related to glucose tolerance, insulin sensitivity and dyslipidemia in rats.

**Methods:**

Forty male Wistar rats were randomly assigned to the following dietary treatments (n = 10/group), for 60 days: 1) Normal fat-Soybean oil (NF-So): diet containing 4.0% soybean oil (SO); 2) High Fat-Control Butter (HF-Cb): diet containing 21.7% control butter and 2.3% SO; 3) High Fat-CLA enriched Butter (HF-CLAb): diet containing 21.7% *cis*-9, *trans*-11 CLA-enriched butter and 2.3% SO; and 4) High fat-Soybean oil (HF-So): diet containing 24.0% SO. HF-Cb and HF-CLAb diets contained 0.075% and 0.235% of *cis*-9, *trans*-11 CLA, respectively.

**Results:**

HF-CLAb-fed rats had lower serum insulin levels at fasting than those fed with the HF-Cb diet, while the PPARγ protein levels in adipose tissue was increased in HF-CLAb-fed rats compared to HF-Cb-fed rats. Furthermore, R-QUICK was lower in HF-Cb than in NF-So group, while no differences in R-QUICK were observed among NF-So, HF-CLAb and HF-So groups. Serum HDL cholesterol levels were higher in HF-CLAb-fed rats than in those fed NF-So, HF-Cb and HF-So diets, as well as higher in NF-So-fed rats than in HF-Cb and HF-So-fed rats. HF-CLAb, HF-Cb and HF-So diets reduced serum LDL cholesterol levels when compared to NF-So, whereas serum triacylglycerol levels were increased in HF-CLAb.

**Conclusion:**

Feeding rats on a high-fat diet containing butter naturally enriched in *cis*-9, *trans*-11 CLA prevented hyperinsulinemia and increased HDL cholesterol, which could be associated with higher levels of *cis*-9, *trans*-11 CLA, vaccenic acid, oleic acid and lower levels of short and medium-chain saturated fatty acids from butter naturally modified compared to control butter. On the other hand CLA-enriched butter also increased serum triacylglycerol levels, which could be associated with concomitant increases in the content of *trans*-9 and *trans*-10 C18:1 isomers in the CLA-enriched butter.

**Electronic supplementary material:**

The online version of this article (doi:10.1186/1476-511X-13-200) contains supplementary material, which is available to authorized users.

## Background

Diabetes mellitus is an important cause of mortality and morbidity worldwide, with harmful effects on life expectancy and health-care costs [1]. According to the World Health Organization [2], type 2 diabetes comprises 90% of the total population with diabetes mellitus around the world, and is characterized by the body’s ineffective use of insulin. It is projected that the number of people with diabetes mellitus worldwide will rise to 439 million by 2030 [3]. There is compelling evidence that diet plays an important role in the prevention of a number of non-communicable diseases, including type-2 diabetes [4]. In this context, conjugated linoleic acid (CLA) has attracted considerable attention in the scientific community due to its health-promoting properties reported in a number of *in vitro* and animal studies [5]. CLA refers to the positional and geometric conjugated dienoic isomers of linoleic acid (C18:2 n-6) [6] which are predominantly found in ruminant fat [7]. Although nearly twenty isomers have been identified in ruminant products [8], 75-90% of total CLA is represented by *cis*-9, *trans*-11 CLA (rumenic acid) [9], whereas the *trans*-10, *cis-* 12 CLA isomer is normally found in very low concentrations [10]. There is some evidence that the anti-diabetogenic effects reported in several studies are mediated by rumenic acid [11], the major CLA isomer in ruminant fat.

As dairy products are the major source of CLA in the human diet [7], efforts have been made to increase the milk fat CLA content, which can be achieved by including plant oils in the diet of dairy cows [12, 13]. Most of the *cis*-9, *trans*-11 CLA secreted in milk is synthesized endogenously from trans-11 C18:1 (vaccenic acid) through stearoyl-CoA desaturase enzyme (also known as Δ-9 desaturase). Therefore, milk naturally enriched in *cis*-9, *trans*-11 CLA is also a rich source of vaccenic acid [14]. Endogenous synthesis of *cis*-9, *trans*-11 CLA from C18:1 *trans*-11 has also been reported in humans [15] and other species [16, 17], which further contributes to increasing the *cis*-9, *trans*-11 CLA levels in the body tissues. It should also be noted that the concentrations of some minor (e.g. *trans*-C18:1 isomers other than vaccenic) and major (e.g. medium-chain saturated) fatty acids are also altered in milk fat from cows fed diets supplemented with plant oils [13], which should be taken into account when food sources naturally enriched in CLA are used in a given study.

In light of the potential anti-diabetogenic effects of *cis*-9, *trans*-11 CLA observed in previous studies and the shortage of studies addressing CLA from natural source, we investigated the effects of a diet containing butter naturally enriched in *cis*-9 *trans*-11 CLA on glucose tolerance, insulin sensitivity and dyslipidemia in Wistar rats.

## Results

**Figure 1:**
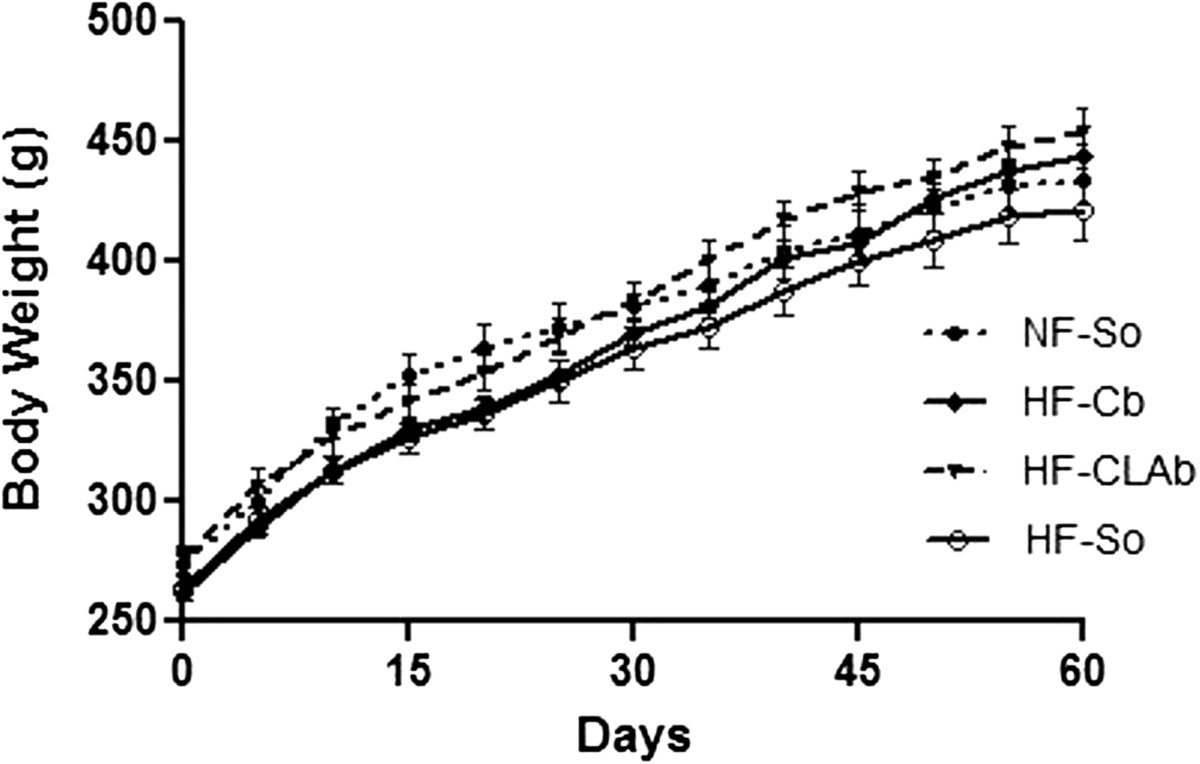
**Effect of control or naturally enriched in** ***cis*** **-9,** ***trans*** **-11 CLA butters on body weight.** Male Wistar rats fed the following dietary treatments for 60 days: Normal fat-Soybean oil (NF-So): diet containing 4.0% soybean oil (SO); High Fat-Control Butter (HF-Cb): diet containing 21.7% control butter and 2.3% SO; High Fat-CLA enriched Butter (HF-CLAb): diet containing 21.7% *cis*-9, *trans*-11 CLA-enriched butter and 2.3% SO; High fat-Soybean oil (HF-So): diet containing 24.0% SO. All data are presented as mean values ± S.E.M (n = 10 rats/group). Statistically significant differences were determined by Anova followed by Newman-Keuls. *p < 0.05, **p <0.01.

Food intake of HF-Cb, HF-CLAb and HF-So diets was 20.76%, 19.54% and 27.60% lower than NF-So food intake, respectively, while no difference was observed between HF-Cb, HF-CLAb and HF-So (Table 1). The energy intake observed in rats fed with the HF-Cb, HF-CLAb and HF-So diets was 15.85%, 13.95% and 11.04% higher than in NF-So-fed rats, respectively, but there was no difference among HF-Cb, HF-CLAb and HF-So (Table 1). No differences in weight gain (expressed as a percentage of initial weight) were observed among treatment groups (Table 1). The effect of NF-So, HF-Cb, HF-CLAb and HF-So diets on body weight during all experimental period is shown in Figure 1. There were no differences among dietary treatments.

**Table 1:** **Metabolic and serum parameters in Wistar rats fed with control or naturally enriched in** ***cis*** **-9,** ***trans*** **-11 CLA butters for 60 days**

	Dietary treatments			
	NF-So^1^	HF-Cb^2^	HF-CLAb^3^	HF-So^4^
**Dietary intake and weight gain**				
**Intake (g/day/rat)**	26.45 ± 1.06	20.96 ± 0.37***	21.33 ± 0.49***	19.15 ± 0.49***
**Intake (Kcal/day/rat)**	63.19 ± 2.52	73.21 ± 1.31**	72.01 ± 1.67**	70.17 ± 1.89*
**Weight gain (%)**	62.15 ± 1.90	69.31 ± 2.13	66.05 ± 2.41	59.80 ± 3.32
**Body composition**				
**Moisture (%)**	50.10 ± 1.05	50.03 ± 0.47	48.19 ± 0.44	50.83 ± 1.17
**Lipid (%)**	29.41 ± 1.38	28.55 ± 0.64	31.31 ± 0.50	27.14 ± 1.36
**Protein (%)**	17.76 ± 0.32	17.60 ± 0.21	16.96 ± 0.19	17.57 ± 0.49
**Ash (%)**	3.38 ± 0.05	4.13 ± 0.09	3.66 ± 0.28	3.87 ± 0.38
**Insulin Sensibility Indexes and AUC**				
**HOMA index**	1.11 ± 0.02	1.40 ± 0.10	1.39 ± 0.16	1.08 ± 0.05
**R-QUICKI**	0.88 ± 0.02	0.76 ± 0.03*	0.82 ± 0.02	0.81 ± 0.04
**AUC**	13180 ± 1505	12330 ± 1158	14390 ± 1398	14610 ± 1021
**Serum metabolites**				
**NEFA (mmol/L)**	0.375 ± 0.023	0.325 ± 0.017	0.354 ± 0.022	0.294 ± 0.025
**Leptin (ng/mL)**	2.21 ± 0.21	2.59 ± 0.26	2.72 ± 0.35	1.99 ± 0.20
**LDL-C** ^**5**^ **/HDL-C** ^**6**^	1.42 ± 0.07	0.93 ± 0.04***^,##^	0.81 ± 0.05^***,###^	1.17 ± 0.06**
**non-HDL-C/HDL-C**	1.73 ± 0.11	1.39 ± 0.08*	1.33 ± 0.07**	1.46 ± 0.05*

Data are presented as mean values ± S.E.M (n = 10 rats/group). Statistically significant differences were determined by Anova followed by Newman-Keuls. Asterisk denotes statistically significant differences compared to NF-So (*p < 0.05, **p < 0.01, ***p < 0.001) and number sign denotes statistically significant differences compared to HF-So (^##^*p* < 0.01, ^###^*p* < 0.001). ^1^Normal Fat-Soybean oil (NF-So), diet containing 4.0% soybean oil (SO); ^2^High Fat-Control Butter (HF-Cb), diet containing 21.7% control butter and 2.3% SO; ^3^High CLA Butter (HF-CLAb), diet containing 21.7% butter naturally enriched in *cis*-9, *trans*-11 CLA and 2.3% SO; ^4^High Fat-Soybean oil (HF-So), diet containing 24.0% SO.

^5^LDL-C: LDL cholesterol; ^6^HDL-C:HDL cholesterol.

**Figure 2:**
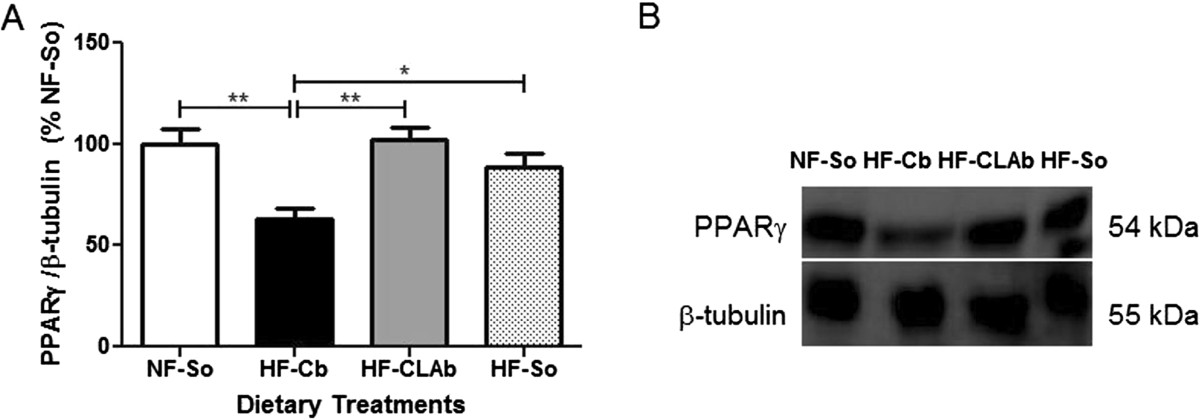
**Analysis of PPARγ protein level in retroperitoneal adipose tissue.** PPARγ levels **(A)** and representative blot for PPARγ and β-tubulin (loading control) (additional Electrophoretic blot files show this in more detail [see Additional files 1, 2, 3 and 4]) **(B)** of male Wistar rats fed the following dietary treatments for 60 days: Normal fat-Soybean oil (NF-So): diet containing 4.0% soybean oil (SO); High Fat-Control Butter (HF-Cb): diet containing 21.7% control butter and 2.3% SO; High Fat-CLA enriched Butter (HF-CLAb): diet containing 21.7% *cis*-9, *trans*-11 CLA-enriched butter and 2.3% SO; High fat-Soybean oil (HF-So): diet containing 24.0% SO. All data are presented as mean values ± S.E.M (n = 10 rats/group). Statistically significant differences were determined by Anova followed by Newman-Keuls. *p <0.05, **p <0.01.

Concerning the carcass chemical composition, no differences in moisture, lipid, protein and ash contents was observed among groups (Table 1). PPARγ protein levels in adipose tissue were decreased by 58.70%, 62.35% and 41% in HF-Cb-fed rats in comparison to those fed with the NF-So, HF-CLAb and HF-So diets, respectively (Figure 2) (Additional files 1, 2, 3 and 4).

**Figure 3:**
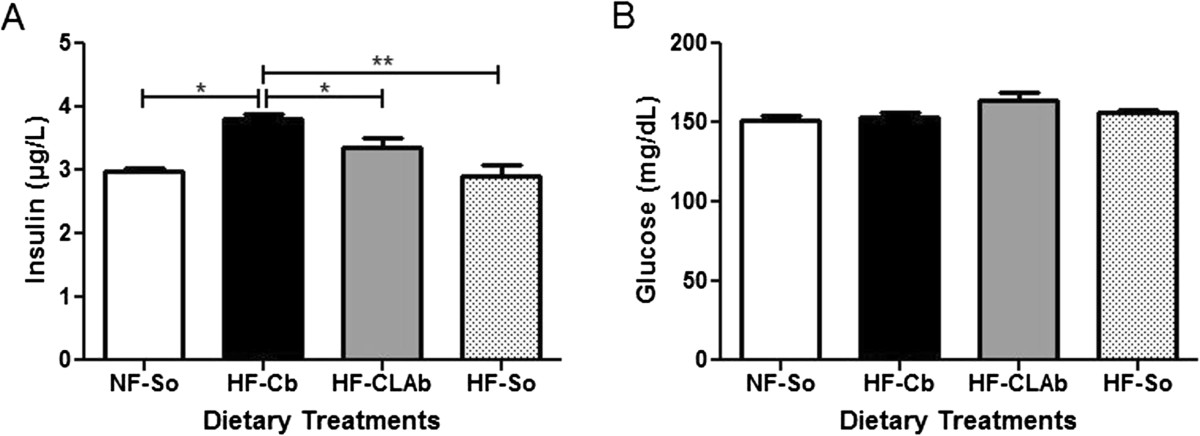
**Effects of control or naturally enriched in** ***cis*** **-9,** ***trans*** **-11 CLA butters on serum metabolites.** Insulin **(A)** and glucose **(B)** of male Wistar rats fed the following dietary treatments for 60 days: Normal fat-Soybean oil (NF-So): diet containing 4.0% soybean oil (SO); High Fat-Control Butter (HF-Cb): diet containing 21.7% control butter and 2.3% SO; High Fat-CLA enriched Butter (HF-CLAb): diet containing 21.7% *cis*-9, *trans*-11 CLA-enriched butter and 2.3% SO; High fat-Soybean oil (HF-So): diet containing 24.0% SO. All data are presented as mean values ± S.E.M (n = 10 rats/group). Statistically significant differences were determined by Anova followed by Newman-Keuls. *p <0.05, **p <0.01.

Fasting serum insulin levels increased by 21.73%, 11.60% and 23.65% in HF-Cb-fed rats in comparison to those fed with the NF-So, HF-CLAb and HF-So diets, respectively (Figure 3A), whereas there were no differences in glycemia levels among experimental groups (Figure 3B). NEFA and leptin did not differ among dietary treatments (Table 1).

**Figure 4:**
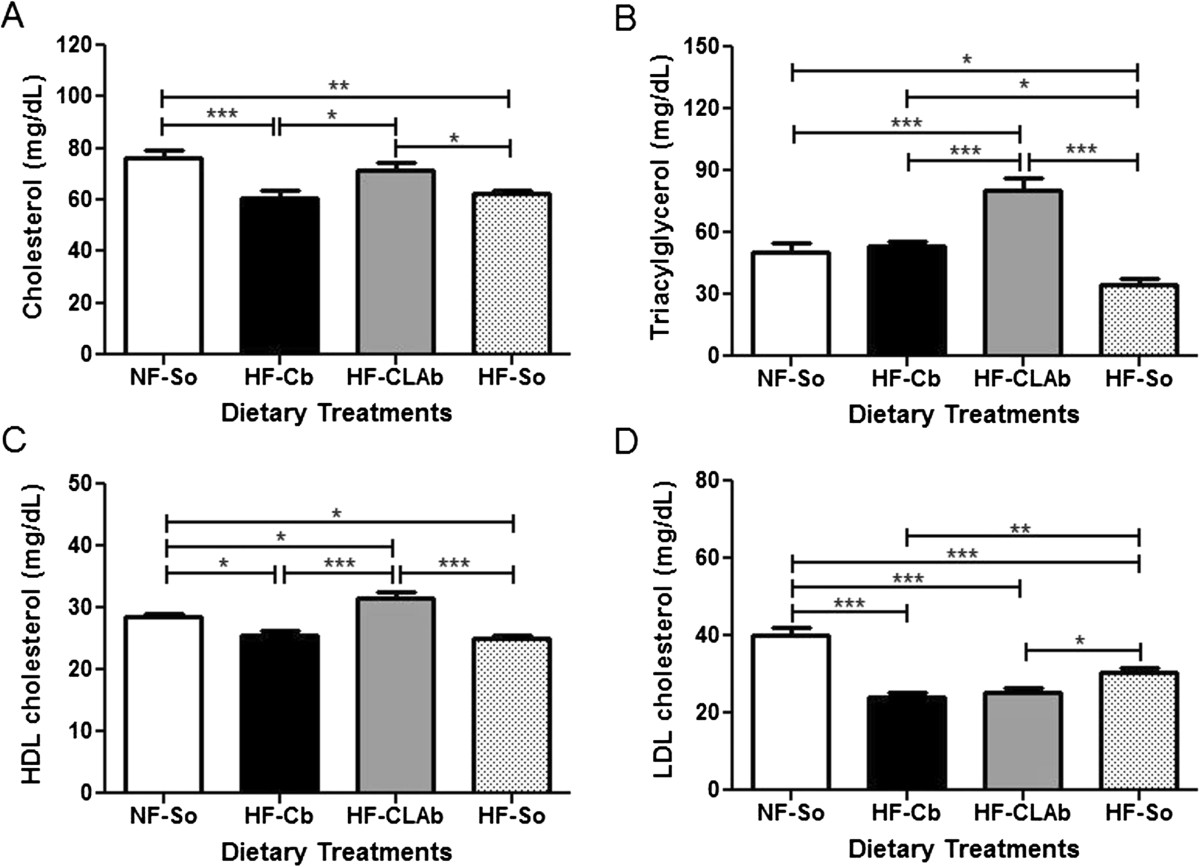
**Effects of control or naturally enriched in** ***cis*** **-9,** ***trans*** **-11 CLA butters on lipid serum.** Cholesterol **(A)**, triacylglycerol **(B)**, HDL cholesterol **(C)** LDL cholesterol **(D)** of male Wistar rats fed the following dietary treatments for 60 days: Normal fat-Soybean oil (NF-So): diet containing 4.0% soybean oil (SO); High Fat-Control Butter (HF-Cb): diet containing 21.7% control butter and 2.3% SO; High Fat-CLA enriched Butter (HF-CLAb): diet containing 21.7% *cis*-9, *trans*-11 CLA-enriched butter and 2.3% SO; High fat-Soybean oil (HF-So): diet containing 24.0% SO. All data are presented as mean values ± S.E.M (n = 10 rats/group). Statistically significant differences were determined by Anova followed by Newman-Keuls. *p <0.05, **p <0.01, ***p <0.001.

HOMA index was unchanged by the dietary treatments (Table 1). However, the HF-Cb group had a lower R-QUICKI index (13.63%) than NF-So, while no difference was observed among HF-CLAb, HF-So and NF-So groups (Table 1). There were no differences in the area under the OGTT glycemic curve (AUC) among dietary treatments (Table 1). Serum cholesterol levels did not differ between HF-CLAb and NF-So groups, whereas there were no differences between HF-Cb and HF-So (Figure 4A). Serum triacylglycerol levels in HF-CLAb were increased by 58.81%, 49.54% and 131.12% when compared to NF-So, HF-Cb and HF-So groups, respectively (Figure 4B). Serum levels of HDL cholesterol were increased by 10.08%, 23.29% and 25.76% in HF-CLAb-fed rats as compared to those fed with the NF-So, HF-Cb and HF-So diets, respectively (Figure 4C). There was no difference in serum LDL cholesterol levels between rats fed with the HF-Cb and HF-CLAb diets, but values observed in these groups were 39.68% and 36.88% lower than in NF-So group, respectively, and 21.05% and 17.37% lower than in HF-So, respectively (Figure 4D). There was no difference in the LDL cholesterol:HDL cholesterol ratio between HF-Cb and HF-CLAb groups, and these values were lower than HF-So result. The LDL cholesterol:HDL cholesterol ratio of high fat diet groups were lower than the value of NF-So (Table 1). There was no difference in the non-HDL cholesterol:HDL cholesterol ratio among HF-Cb, HF-CLAb and HF-So groups, while these values were lower than NF-So result (Table 1).

## Discussion

In recent years, conjugated linoleic acid has received much attention as a dietary supplement [11], however few studies assess the effects of CLA from natural sources on insulin, glucose and serum lipid metabolism. In this paper, we have demonstrated dietary effects of *cis*-9, *trans*-11 CLA-enriched butter in 60-day-old Wistar rats on feed intake, body composition, insulin and glucose metabolism as well as dyslipidemia.

In this study, there were no differences in dietary intake among rats fed with *cis*-9, *trans*-11 CLA-enriched butter, control butter or high fat-soybean oil. HF-Cb, HF-CLAb or HF-So-fed rats adapted to the higher energy density of these diets by reducing their daily food intake compared to the NF-So group, as was previously reported [18]. Daily energy intake was higher in HF-Cb, HF-CLAb and HF-So-fed rats than in the NF-So group, which can be attributed to the increased palatability of high fat diets, which is directly related to higher energetic intake [19]. High fat diets are more palatable because fat content is one of the factors that contribute to food palatability [19].

Experiments have shown that PPARγ is the master adipogenic regulator [20] and, interconnected to its role in adipocyte differentiation, PPARγ regulates insulin sensitivity by transcriptionally activating genes involved in insulin signaling, glucose uptake, and fatty acid uptake and storage [21]. HF-CLAb-fed rats presented increased levels of PPARγ in adipose tissue compared to HF-Cb-fed rats, which may be attributed to higher (213.20%) supply of *cis*-9, *trans*-11 CLA from the CLA-enriched butter diet in comparison to the control butter diet. Studies have demonstrated that *cis*-9, *trans-* 11 CLA increased the expression of PPARγ, whose down-regulation may lead to insulin resistance [22]. It was demonstrated that CLA mixed with 0.286% *cis*-9, *trans*-11 CLA increased the mRNA expression of PPARγ in adipose tissue of Wistar rats, which was related to improved insulin sensitivity [23]. Besides, it was shown that depletion of PPARγ in adipose tissue causes insulin resistance, since decreased PPARγ action in mature adipocytes, leads to reduced expression of key genes required for insulin signaling in adipocytes [24]. It was previously shown that adipocyte-specific constitutive activation of PPARγ in mature adipocytes can regulate whole body insulin sensitivity [25].

Therefore, CLA-enriched butter was shown as having action mechanisms PPARγ-dependent, up-regulating its expression in adipose tissue, and preventing PPARγ reduction as was observed by a control butter diet.

Rats fed with *cis*-9, *trans*-11 CLA-enriched butter had lower fasting serum insulin levels than rats fed with control butter. Therefore HF-CLAb diet prevented the fasting hyperinsulinemia, which is a result potentially beneficial. According to the European Group for the Study of Insulin Resistance, fasting insulin is the best available simple proxy for insulin resistance, which is defined by presence of fasting hyperinsulinemia [26]. Besides, it was demonstrated that a gradual increase in serum insulin in the fasting state reflects decreased insulin sensitivity [27]. HOMA index did not differ among experimental groups, however R-QUICKI index, which also denotes insulin sensitivity [28], was lower in the HF-Cb group compared to the NF-So group, while there was no difference among the NF-So, HF-CLAb and HF-So groups. Thus, R-QUICKI index shows that control butter diet induces insulin resistance compared to normal fat diet, a condition that was not observed in HF-CLAb group and may be associated to PPARγ reduced level in adipose tissue of HF-Cb-fed rats [24].

The beneficial effect of *cis*-9, *trans*-11 CLA-enriched butter on fasting insulin level might be due to the higher supply of *cis*-9, *trans*-11 CLA from the CLA-enriched butter diet in comparison to the control butter diet. It was previously shown that animals fed with a 0.25% *cis*-9, *trans*-11 CLA diet decreased serum insulin concentration at fasting [11]. As observed in Table 1, the concentrations of several fatty acids were also altered in the HF-CLAb diet as compared to the HF-Cb diet. For instance, there was a higher (269.72%) supply of vaccenic acid from HF-CLAb diet compared to HF-Cb diet, which contributed to increase the tissue level of *cis*-9, *trans*-11 CLA in HF-CLAb-fed rats [16]. Furthermore, there was a lower (32.06%) supply of short and medium-chain saturated fatty acids from HF-CLAb diet compared to HF-Cb diet, which could also have contributed to the decreased fasting serum insulin level of the HF-CLAb group, since it has been suggested that diets high in saturated fatty acids have effects on hyperinsulinemia [29–31]. Despite the changed parameters of HF-Cb-fed rats, the areas under the curves of oral glucose tolerance tests did not differ among NF-So, HF-Cb, HF-CLAb and HF-So-fed rats, therefore the experimental diets were not responsible for glucose intolerance.

Serum NEFA concentration is a risk factor for type 2 diabetes because the combination of excessive levels of non-esterified fatty acids and glucose leads to decreased insulin secretion, impairments in insulin gene expression and beta-cell death by apoptosis [32]. Previous studies showed that *cis*-9, *trans-* 11 CLA reduced NEFA levels [11] however, in the present investigation, there were no differences among groups. The lack of an effect of butter enriched in *cis*-9, *trans*-11 CLA on NEFA may be attributed to altered bioavailability and bioactivity of *cis*-9, *trans*-11 CLA when inserted into the fat butter. A similar hypothesis was developed when it was observed less distinct effect of high-CLA beef compared to synthetic CLA on the proteome of insulin-sensitive tissues [33]. Leptin is an adipokine that plays a role in glucose metabolism and insulin sensitivity [34], however in the present study there were no differences among groups. Similarly, it was shown in previous studies that *cis*-9, *trans-* 11 CLA did not alter leptin levels [11, 22, 35].

In the present work, serum cholesterol and LDL cholesterol concentrations were not modified by the HF-CLAb diet compared to the NF-So and HF-Cb diets, respectively. Similarly, no effects of *cis*-9, *trans*-11 CLA on cholesterol and LDL cholesterol levels were also shown previously [36, 37]. The high LDL cholesterol concentration in NF-So-fed rats may be due to high levels of carbohydrate (73.39% of energy) in this diet, since it was demonstrated that when dietary carbohydrate was increased from 50% to 67% of energy, the fasting triacylglycerol level rose [38], which is commonly related to increased precursors of LDL cholesterol in the blood, the very-low-density lipoproteins, and consequently increased LDL cholesterol levels [39]. Decreased total cholesterol concentration in HF-Cb or HF-So-fed rats was related to the low HDL cholesterol level in these groups, which is a risk factor for type 2 diabetes mellitus [40].

Increased triacylglycerol levels in HF-CLAb-fed rats may be due to higher (160.37%) contents of *trans*-9 and *trans*-10 C18:1 isomers in the HF-CLAb diet compared to the HF-Cb diet. It has been shown that high intake of *trans*-9 C18:1 was correlated to increased plasma concentration of triacylglycerol [41] as well as the high intake of *trans*-10 C18:1 [42]. Concerning the effect of *cis*-9, *trans*-11 CLA on the triacylglycerol level, previous studies in animals fed with this CLA isomer did not modify triacylglycerol concentration [43, 44]. However, rats fed with the HF-CLAb diet had an increased HDL cholesterol level, which is a potentially beneficial result because it reduces the risk of having a cardiovascular event [45] and HDL cholesterol also has a positive effect on glycemic control [45]. The high level of HDL cholesterol in HF-CLAb-fed rats may be attributed to a higher level of *cis*-9, *trans*-11 CLA, as also reported by a previous study [46]. Similarly, it was demonstrated that high CLA enriched clarified butter increased plasma HDL cholesterol in Wistar rats [47]. However, it is possible that the higher supply of oleic acid (*cis*-9 C18:1) (27,61%) from the HF-CLAb diet compared to the HF-Cb diet may also have contributed to increased HDL cholesterol levels, since it has been suggested that oleic acid has effects on increasing HDL cholesterol [48]. Besides, there was a lower (36.91%) supply of lauric (C12:0) and myristic (C14:0) acids from HF-CLAb diet than HF-Cb diet, which could also have contributed to raised HDL cholesterol levels of HF-CLAb group, since it was demonstrated that a lauric and myristic acid-rich diet decreased HDL cholesterol concentration [49]. On the other hand, the HF-CLAb diet had higher (147.82%) levels of *trans* **-** 9 C18:1, which has been associated with decreased levels of HDL cholesterol [50]. Therefore, we hypothesized that fatty acids related to increased HDL cholesterol level were capable of acting synergistically, prevailing over negative effects of *trans* **-** 9 C18:1 isomers on HDL cholesterol levels, resulting in higher concentration of this lipoprotein in HF-CLAb-fed rats. However, concerning the triacylglycerol levels, it has already been demonstrated by a previous study with animals fed with butter naturally enriched in *cis*-9 *trans*-11 CLA that this diet had no effect on the plasma concentration of triacylglycerol [14]. Thus, it was possible to hypothesize that the higher contents of *trans* **-** 9 and *trans* **-** 10 C18:1 isomers in the HF-CLAb diet prevailed over the absence of *cis*-9 *trans*-11 CLA effects on triacylglycerol levels, resulting in a higher concentration of triacylglycerol in HF-CLAb-fed rats.

## Conclusion

In conclusion, the present investigation suggests that a 60 day feeding of a diet containing butter naturally enriched in *cis*-9, *trans*-11 CLA to 60-day-old male Wistar rats has effects on insulin, HDL cholesterol and triacylglycerol metabolism. *Cis*-9, *trans*-11 CLA-enriched butter significantly raised serum HDL cholesterol and prevented fasting hyperinsulinemia, which could be attributed to higher levels of *cis*-9, *trans*-11 CLA, vaccenic acid, oleic acid and lower levels of short and medium-chain saturated fatty acids from CLA-enriched butter compared to control butter. However, CLA-enriched butter was also found to cause fasting hypertriglyceridemia, which could be associated with concomitant increases in the content of *trans* **-** 9 and *trans* **-** 10 C18:1 isomers in the CLA-enriched butter. Additional studies are still needed before conjugated linoleic acid from natural sources can be used in human diets as a functional food to decrease type-2 diabetes risk factors.

## Methods

### Ethics statement

This study was carried out in strict accordance with the recommendations of the Guide for the Care and Use of Laboratory Animals [51]. All procedures with animals were approved by the Ethic Committee on Animal Experimentation of Federal University of Juiz de Fora at Minas Gerais, Brazil, protocol number 054/2012.

### Animals

Forty (n = 40) male Wistar rats (*Rattus norvegicus Berkenhout, 1769*), 60 days old and weighing 250–300 g, were obtained from the Center of Reproduction Biology of the Federal University of Juiz de Fora, Minas Gerais, Brazil. They were kept in a controlled temperature environment (23 ± 2°C) with a photoperiod of 12 hours (7 a.m. to 7 p.m. - light and 7 p.m. to 7 a.m. - dark). Water and the experimental diets were offered on an *ad libitum* basis to the animals throughout the study.

### Production of experimental butters

Experimental butters used in the current study were produced at Embrapa Dairy Cattle (Juiz de Fora, Minas Gerais, Brazil). Standard butter and *cis*-9, *trans*-11 CLA-enriched butter were produced from milk of cows (Holstein x Gir) fed diets composed of either corn silage and concentrate containing no sunflower oil, or chopped elephant grass and concentrate supplemented with sunflower oil at 4.5% of diet dry matter, respectively. The butters were produced as described previously [52].

### Dietary treatments and experimental design

After a 7 day acclimatization period in which all animals were fed a commercial chow (Nuvital, Colombo, PR, Brazil), the rats were randomly assigned to four dietary treatments (n = 10/group), for 60 days: 1) Normal fat-Soybean oil (NF-So): diet containing 4.0% soybean oil (SO); 2) High Fat-Control Butter (HF-Cb): diet containing 21.7% control butter and 2.3% SO; 3) High Fat-CLA enriched Butter (HF-CLAb): diet containing 21.7% *cis*-9, *trans*-11 CLA-enriched butter and 2.3% SO; and 4) High fat-Soybean oil (HF-So): diet containing 24.0% SO. SO was included in both HF-Cb and HF-CLAb diets in order to reach the requirements of linoleic and linolenic acids to adults rats [53].

All diets were produced according to the American Institute of Nutrition (AIN-93 M) [53]. Ingredients were carefully mixed in order to obtain a homogeneous mass which was used to produce handmade pellets. The pellets were prepared weekly, purged with nitrogen and stored at -20°C in daily portions in sealed polythene bags to minimize the oxidation of fatty acids. The composition of purified diets is presented in Table 2.

**Table 2:** **Ingredient composition of experimental diets**

Ingredient	% of the diet (g/100 g of diet)
**Corn starch** ^**2**^	46.6 or 29.1^a,b,c,d^
**Dextronized corn starch** ^**2**^	15.5
**Casein** ^**1**^	14.0 or 17.3^a,b,c,d^
**Sucrose** ^**1**^	10.0
**Cellulose** ^**2**^	5.0
**AIN-93 mineral mix** ^**1**^	3.5
**AIN-93 vitamin mix** ^**1**^	1.0
**L-Cystine** ^**2**^	0.18
**Choline bitartrate** ^**2**^	0.25
**tert-Butylhydroquinone** ^**1**^	0.01
**SO**^**3**^**or Butter**^**4**^ **+ SO**^**a,b,c**^	4.0 or 24.0^a,b,c,d^

^1,2^Dietary ingredients were purchased from Rhoster (Araçoiaba da Serra, SP, Brazil) and Farmos (Rio de Janeiro, RJ, Brazil); ^3^Soybean oil (SO); ^4^Control Butter or High CLA Butter. ^a^Normal Fat-Soybean diet consisted of 46.6% corn starch, 14.0% casein and 4.0% SO; ^b^High Fat-Control butter diet consisted of 29.1% corn starch, 17.3% casein and 21.7% Standard Butter + 2.3% SO; ^c^High Fat-CLA enriched butter diet consisted of 29.1% corn starch, 17.3% casein and 21.7% High CLA Butter + 2.3% SO; ^d^High Fat-Soybean oil diet consisted of 29.1% corn starch, 17.3% casein and 24.0% SO.

Samples of pellets (50 g) from each diet were randomly collected and analyzed for chemical composition according to reference methods [54, 55]. To determine the fatty acid composition of experimental diets, total lipids were extracted according to Hara and Radin [56] using a 3:2 (vol:vol) mixture of hexane and isopropanol (4.5 mL/g of pellet) followed by a 67 g/L of sodium sulfate solution (3 mL/g of pellet). Fatty acid methyl esters (FAME) were obtained by base-catalyzed transmethylation using a freshly prepared methylation reagent (0.4 mL of 5.4 mol/L of sodium methoxide solution + 1.75 mL of methanol) according to Christie et al., [57] with modifications [58]. The mixture was neutralized with oxalic acid (1 g of oxalic acid in 30 mL diethyl ether) and calcium chloride was added to remove methanol residues. The FAME were determined by gas chromatography (model 6890 N; Agilent Technologies Brasil Ltda., Barueri, Brazil) fitted with a flame-ionization detector and equipped with a CP-Sil 88 fused silica capillary column (100 m × 0.25 mm × 0.2 μm film thickness; Varian Inc., Mississauga, ON). Operating conditions included injector and detector temperatures both at 250°C, H_2_ as the carrier gas (1 mL/min), and for the flame-ionization detector (35 mL/min), N_2_ as the makeup gas (30 mL/min), and purified air (286 mL/min). The initial temperature was 45°C and held for 4 min, increased by 13°C/min to 175°C and held for 27 min, and increased by 4°C/min to 215°C and held for 35 min [59]. The FAME were identified by comparison with 4 FAME reference standards (Supelco37 mix #47885-U, linoleic acid isomers mix #47791, CLA isomers mix #05632; Sigma-Aldrich, St. Louis, MO, and Nu-Chek GLC-463); minor *trans*-18:1 isomers were identified according to their elution order reported under the same chromatographic conditions [59, 60]. The fatty acid composition of experimental diets was expressed as a weight percentage of total fatty acids using theoretical relative response factors described by Wolff et al., [61] (Table 3).

**Table 3:** **Chemical composition and fatty acid profile of the experimental diets**

	Dietary treatments			
	NF-So^2^	HF-Cb^3^	HF-CLAb^4^	HF-So^5^
	**Chemical composition,% of diet dry matter**			
**Dry matter content (%)**	79.1	86.8	85.4	88.4
**Fat**	3.11	17.6	17.4	21.1
**Crude protein**	13.1	16.0	16.2	14.8
**Ash**	2.76	2.98	3.09	2.95
**Neutral Detergent Fiber (NDF)**	2.76	3.55	3.26	3.89
**Carbohydrate**	55.4	44.8	43.4	42.7
	**Energetic composition**			
**Carbohydrate Energy (%)**	73.4	44.6	43.9	40.7
**Protein Energy (%)**	17.4	15.9	16.4	14.1
**Fat Energy (%)**	9.35	39.5	39.6	45.2
**Kcal/g**	2.39	3.49	3.38	3.71
	**Fatty acids (g/100 g of total fatty acids)**			
**C4:0**	n.d.^1^	3.16	2.95	n.d.
**C5:0**	n.d.	0.03	0.01	n.d.
**C6:0**	n.d.	1.69	1.37	n.d.
**C7:0**	n.d.	0.02	0.01	n.d.
**C8:0**	n.d.	1.00	0.64	n.d.
**C9:0**	n.d.	0.03	0.01	n.d.
**C10:0**	n.d.	2.07	1.14	n.d.
**C10:1** ***cis*** **-9**	n.d.	0.26	0.12	n.d.
**C11:0**	n.d.	0.02	0.01	n.d.
**C12:0**	n.d.	2.37	1.25	n.d.
**C12:1** ***cis*** **-9/C13:0**	n.d.	0.16	0.08	n.d.
**C14:0**	0.52	8.71	5.74	0.54
**C15:0** ***iso***	n.d.	0.20	0.25	n.d.
**C15:0** ***anteiso***	n.d.	0.41	0.47	n.d.
**C14:1** ***cis*** **-9**	n.d.	0.83	0.46	n.d.
**C15:0**	n.d.	0.95	0.90	n.d.
**C16:0**	11.7	29.3	19.7	11.8
**C16:1** ***trans*** **-9**	n.d.	0.03	0.03	n.d.
**C17:0** ***iso***	n.d.	0.32	0.51	n.d.
**C16:1** ***cis*** **-9 + C17:0** ***anteiso***	n.d.	1.51	1.16	n.d.
**C17:0**	n.d.	0.49	0.51	n.d.
**C17:1** ***cis*** **-9**	n.d.	0.18	0.19	n.d.
**C18:0**	4.25	9.02	13.9	4.23
**C18:1** ***trans*** **-4**	n.d.	0.02	0.07	n.d.
**C18:1** ***trans*** **-5**	n.d.	0.02	0.06	n.d.
**C18:1** ***trans*** **-6/7/8**	n.d.	0.31	0.80	n.d.
**C18:1** ***trans*** **-9**	n.d.	0.23	0.57	n.d.
**C18:1** ***trans*** **-10**	n.d.	0.30	0.81	n.d.
**C18:1** ***trans*** **-11**	n.d.	1.09	4.03	n.d.
**C18:1** ***trans*** **-12**	n.d.	0.29	0.65	n.d.
**C18:1** ***trans*** **-13/14**	n.d.	0.24	0.49	n.d.
**C18:1** ***cis-*** **9/** ***trans*** **-15**	23.8	20.3	25.9	22.4
**Minor** ***cis*** **-C18:1 isomers (c11 + c12 + c13)**	1.43	0.83	1.03	1.45
**C18:1** ***trans*** **-16**	n.d.	0.23	0.36	n.d.
**C18:1** ***cis*** **-14**	n.d.	0.05	0.10	n.d.
**C19:0/C18:1** ***cis*** **-15**	n.d.	0.11	0.11	n.d.
**C18:2** ***trans*** **-9** ***trans*** **-12**	n.d.	0.01	0.01	n.d.
**C18:2** ***cis*** **-9** ***trans*** **-12**	0.09	0.04	0.06	0.08
**C18:2** ***trans*** **-9** ***cis*** **-12**	n.d.	0.03	0.04	n.d.
**C18:2** ***cis*** **-9** ***cis*** **-12**	49.5	8.04	7.15	52.4
**C20:0**	0.36	0.18	0.20	0.35
**C18:3** ***cis*** **-6,** ***cis*** **-9** ***cis*** **-12**	n.d.	0.02	0.01	n.d.
**C20:1** ***cis*** **-11**	n.d.	0.06	0.12	n.d.
**C18:3** ***cis*** **-9** ***cis*** **-12** ***cis*** **-15**	6.16	0.96	0.89	6.58
**CLA** ***cis*** **-9** ***trans*** **-11**	n.d.	0.53	1.66	n.d.
**CLA** ***trans*** **-10** ***cis*** **-12**	n.d.	0.01	0.01	n.d.
**CLA** ***trans*** **-11** ***cis*** **-13**	n.d.	0.01	0.02	n.d.
**C21:0**	n.d.	0.03	0.03	n.d.
**C20:2** ***cis*** **-11,** ***cis*** **-14**	n.d.	0.02	0.02	n.d.
**C22:0**	0.41	0.11	0.13	0.30
**C20:3 n-6**	n.d.	0.05	0.04	n.d.
**C20:4 n-6**	n.d.	0.10	0.08	n.d.
**C23:0**	n.d.	0.03	0.01	n.d.
**C20:5 n-3 (EPA)**	n.d.	0.02	0.01	n.d.
**C24:0**	0.15	0.06	0.06	0.16
**C22:5 n3 (DPA)**	n.d.	0.06	0.06	n.d.
**C22:6 n-3 (DHA)**	n.d.	n.d.	n.d.	n.d.

^1^n.d.: not detected; ^2^Normal Fat-Soybean oil (NF-So), diet containing 4.0% soybean oil (SO); ^3^High Fat-Control Butter (HF-Cb), diet containing 21.7% control butter and 2.3% SO; ^4^High CLA Butter (HF-CLAb), diet containing 21.7% butter naturally enriched in *cis*-9, *trans*-11 CLA and 2.3% SO; ^5^High Fat-Soybean oil (HF-So), diet containing 24.0% SO.

The *cis*-9, *trans*-11 CLA content in HF-Cb and HF-CLAb diets was calculated as follows: (dry matter content of the diet) x (fat content x 0.95) x (Concentration of *cis*-9, *trans*-11 CLA in g/100 g of total fatty acids). The 5% discount on fat content was applied to correct for the glycerol concentration in triacylglycerol molecules [62]. Based on the above-mentioned calculations, the *cis*-9, *trans*-11 CLA contents in HF-Cb and HF-CLAb diets were 0.075% and 0.235%, respectively. However, considering that about 11% of vaccenic acid (*trans*-11 C18:1) is endogenously converted into rumenic acid in rodents [16], the increase expected of *cis*-9, *trans*-11 CLA in tissue levels of HF-CLAb-fed rats is approximately 15% higher than the levels in HF-Cb-fed rats. The rats were provided fresh food (F_i_) *ad libitum* daily (between 11 a.m and 12 p.m) and the refusals were weighed the next day (F_f_), immediately before the provision of another F_i_. Average food intake (grams/animal) was estimated as follows: (F_i_ - F_f_)/5 (number of animals per cage). Individual body weight was measured every 5 days throughout the treatment period. After the treatment period, the rats were fasted for 12 hours (7 a.m. to 7 p.m.) and blood samples collected from a tail nick for glycemic determinations using the glucose oxidase method [63]. Immediately after glycemic determinations, animals were anesthetized with an intraperitoneal injection of a xylazine (10 mg/Kg)/ketamine (90 mg/Kg) solution, and euthanized by total exsanguination. Glycemic determinations were performed prior to anesthesia as it was shown to induce hyperglycemia [64]. After euthanasia, blood samples, adipose tissue samples and carcasses were analyzed for parameters related to insulin sensitivity and dyslipidemia in rats.

### Analysis of carcass chemical composition

The carcasses were eviscerated, sliced, stored at -80°C, lyophilized (model Liotop L120; Liobras, São Carlos, Brazil) and minced in a knife-type mill. Carcasses were weighed before and after lyophilization to determine their dry matter contents. Moisture, ash, protein and lipid contents were determined according to reference methods [54]. Protein content was quantified using the Kjeldahl method with Foss equipment (model Kjeltec 8400, Foss, Hillerød, Denmark) and lipid content was determined using the Ankom procedure with an Ankom extractor (model XT10, Ankom Technology, New York, USA).

### Analysis of PPARγ protein level by western blot

Retroperitoneal adipose tissue samples were homogenized in a lysis buffer [Tris–HCl: 50 mM, pH 7.4, Na_4_P_2_O_7_: 30 mM, NP-40: 1%, Triton (1%), SDS: 0.1%, NaCl: 150 mM, EDTA: 5 mM, NaF: 50 mM, plus Na_3_VO_4_: 1 mM and protease inhibitor cocktail (Roche Diagnostics, Mannheim, DE)] using an Ultra-Turrax homogenizer (IKA Werke, Staufen, DE). After centrifugation (7500 × g for 5 min), the homogenates were stored at -20°C until SDS-PAGE assay. The total protein content of homogenate was determined by the BCA protein assay kit (Pierce, Illinois, USA). Contents of peroxisome proliferator-activated receptor (PPAR)γ and β-tubulin (loading control) proteins in the retroperitoneal adipose tissue samples were evaluated by incubating monoclonal primary antibodies (anti-PPARγ and anti-β-tubulin; 1:1000; from Abcam, Cambridge, UK) overnight at 4°C, followed by proper secondary antibody (1 hour; 1:7000 antibody from Sigma-Aldrich Co., Missouri, USA) and streptavidin (1 hour; 1:7000; Zymed, California, USA) incubation. The protein bands were visualized by chemiluminescence with Kit ECL Plus (GE Healthcare Life Sciences, Buckinghamshire, UK) followed by exposure in the ImageQuant™ LAS 500 (GE Healthcare Life Sciences). Area and density of the bands were quantified by Image J software (Media Cybernetics, Maryland, USA). The results were normalized by β-tubulin content and expressed as relative (%) to NF-So group.

### Serum metabolites

Blood samples were collected from euthanized animals by cardiac puncture and centrifuged (5714 × g for 5 min) for serum separation. Serum insulin levels were determined using a rat insulin ELISA kit (Mercodia, Uppsala, Sweden). Serum non-esterified fatty acids (NEFA) levels were analyzed using a colorimetric kit (Randox Laboratories, Antrim, United Kingdom), while leptin was analyzed using a Leptin ELISA kit (R&D Systems, Minneapolis, USA). Serum levels of cholesterol, triacylglycerol, HDL cholesterol and LDL cholesterol were determined by colorimetry using the BT 3000 equipment from Wiener laboratories.

### HOMA and R-QUICKI

Homeostatic Model Assessment (HOMA) index was calculated as follows: [fasting insulin (ng/ml) × fasting glucose (mM)]/22.5. A high HOMA index denotes low insulin sensitivity [65], although it should be acknowledged that the HOMA model has not been validated for use in animal models [66]. The Revised Quantitative Insulin Sensitivity Check Index (R-QUICKI) is another equation to assess insulin sensitivity [28]. This index was calculated as following: [1/log fasting insulin (mU/ml) + log fasting glucose (mg/dl) + log NEFA (mmol/l)] [28].

### Oral glucose tolerance test (OGTT)

After 55 days on the experimental diets, the rats were fasted for 12 hours (7 a.m. to 7 p.m) and received a 50% glucose solution (2 g/kg body weight) by oral gavage [67]. Blood samples were collected from a tail nick for glycemic determinations using the glucose oxidase method [63] at 0, 30, 60, 90, 120 and 240 minutes post gavage. Due to reasons previously described, anesthesia was not used in the OGTT. Changes in blood glucose concentration during the oral glucose tolerance test were evaluated by estimation of the total area under the curve (AUC) calculated as an incremental considering the response from the starting point that was analyzed and using the trapezoidal method [68].

### Statistical analysis

The statistical analyses were performed using Prism 5.0 (GraphPad Software, Inc). Data from different dietary groups were analyzed by one-way ANOVA for overall significance followed by Newman-Keuls’s post-hoc tests to identify differences between treatment groups. Results were expressed as means ± SEM (standard error mean). Treatment effects and differences between means were considered significant when *p* < 0.05.

## Electronic supplementary material

Additional file 1:**Complete electrophoretic blot of representative bands of PPARγ level in adipose tissue of Wistar rats.** Figure containing complete electrophoretic blot of representative bands of PPARγ level shown in Figure 2. (PDF 2 MB)

Additional file 2:**Complete electrophoretic blot of representative bands of PPARγ level in adipose tissue of Wistar rats.** Figure containing complete electrophoretic blot of representative bands of PPARγ level shown in Figure 2. In this file we indicate the experimental group related to each band. (PDF 161 KB)

Additional file 3:**Complete electrophoretic blot of representative bands of β-tubulin (loading control) level in adipose tissue of Wistar rats.** Figure containing complete electrophoretic blot of representative bands of β-tubulin level shown in Figure 2. (PDF 746 KB)

Additional file 4:**Complete electrophoretic blot of representative bands of β-tubulin level (loading control) in adipose tissue of Wistar rats.** Figure containing complete electrophoretic blot of representative bands of β-tubulin level shown in Figure 2. In this file we indicate the experimental group related to each band. (PDF 80 KB)

Below are the links to the authors’ original submitted files for images.

Authors’ original file for figure 1

Authors’ original file for figure 2

Authors’ original file for figure 3

Authors’ original file for figure 4

## Abbreviations

CLA: Conjugated linoleic acid
NF-So: Normal fat-soybean oil
SO: Soybean oil
HF-Cb: High fat-control butter
HF-CLAb: High fat-CLA enriched butter
HF-So: High fat-soybean oil
FAME: Fatty acid methyl esters
PPARγ: Peroxisome proliferator-activated receptor γ
HOMA: Homeostatic model assessment
R-QUICKI: Revised quantitative insulin sensitivity check index
OGTT: Oral glucose tolerance test
AUC: Area under the curve.
