# Supplementary figures and images for: Butter naturally enriched in cis-9, trans-11 CLA prevents hyperinsulinemia and increases both serum HDL cholesterol and triacylglycerol levels in rats
Source: Lipids Health Dis. 2014 Dec 22;13:200. doi: 10.1186/1476-511X-13-200 (PMC4364335; doi:10.1186/1476-511X-13-200)

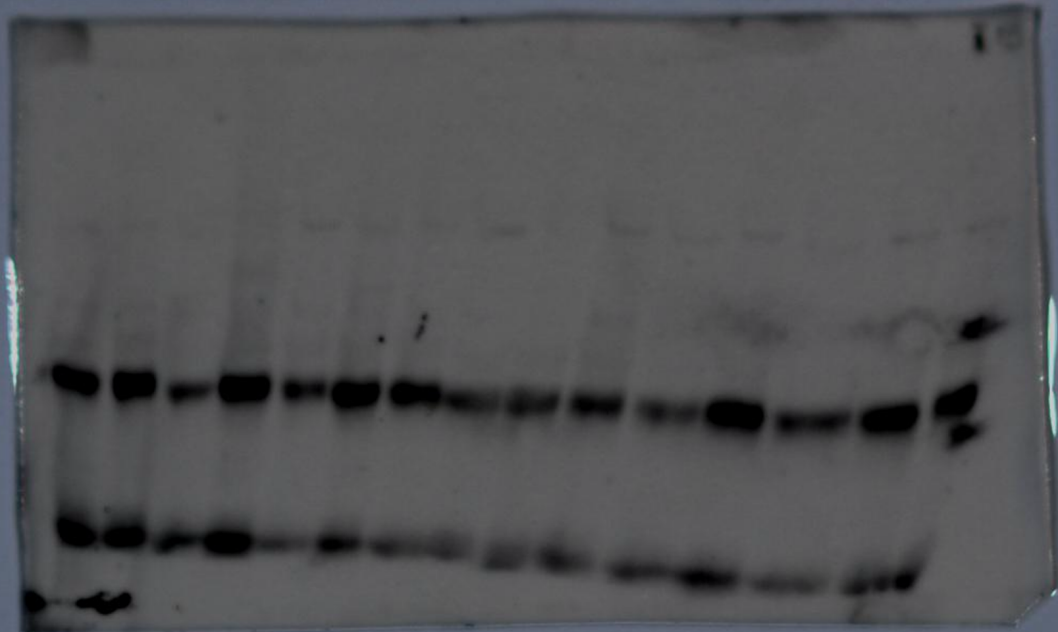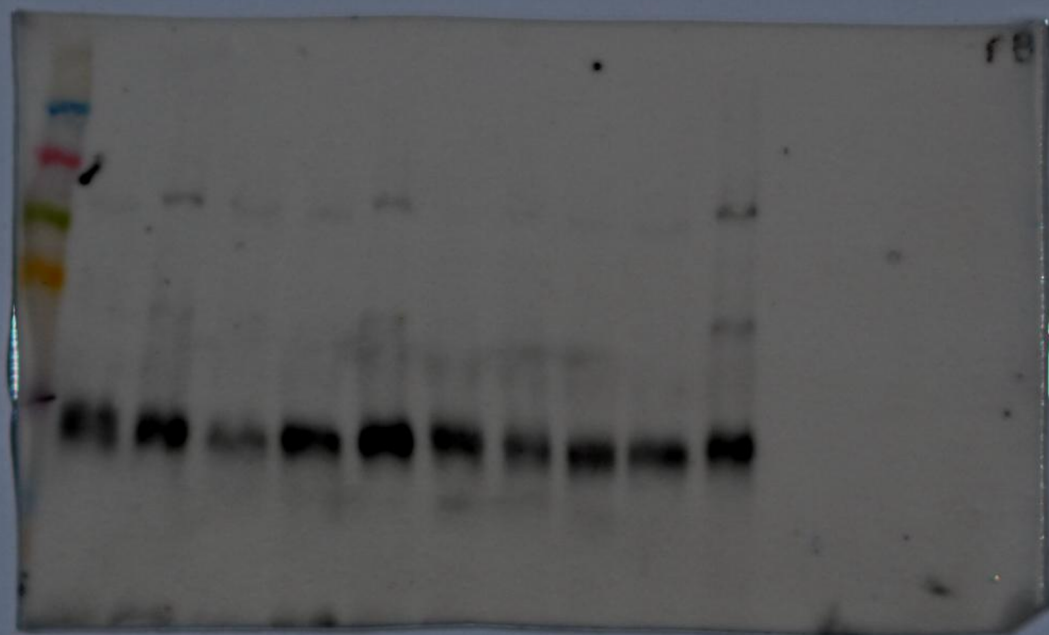

Supplement: Supplementary file 1 — Additional file 1:Complete electrophoretic blot of representative bands of PPARγ level in adipose tissue of Wistar rats. Figure containing complete electrophoretic blot of representative bands of PPARγ level shown in Figure 2. (PDF 2 MB) [file 12944_2014_1191_MOESM1_ESM.pdf]

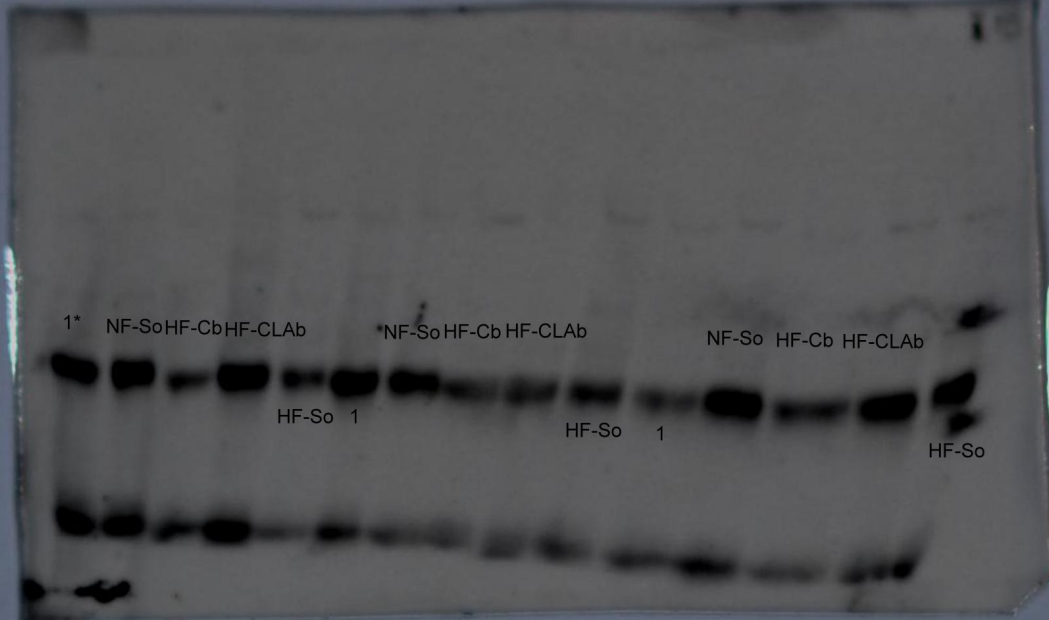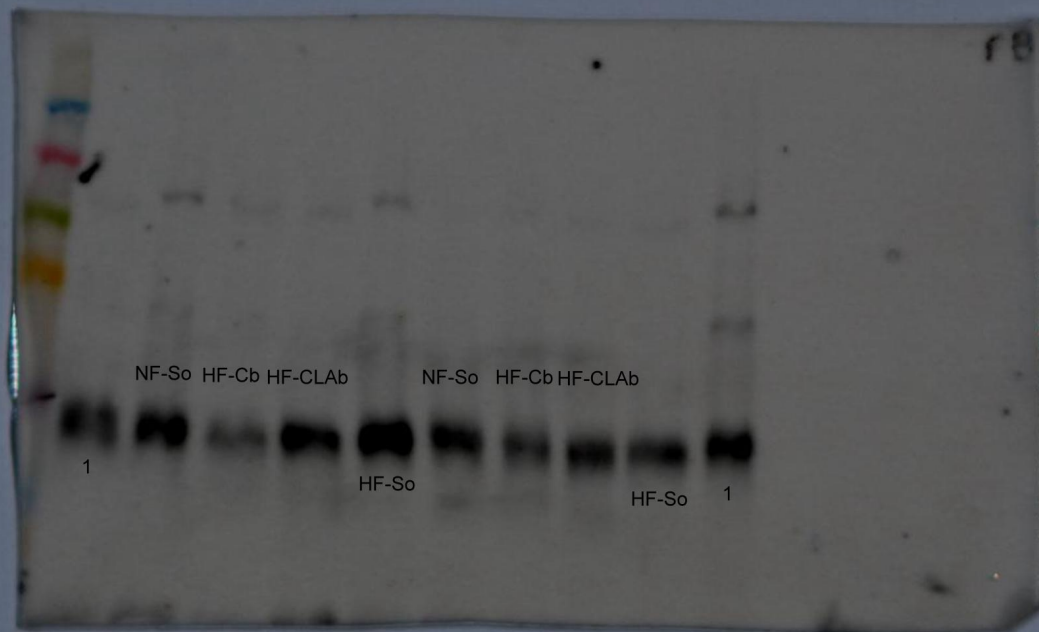

\* 1: Experimental group not addressed in this article

Supplement: Supplementary file 2 — Additional file 2:Complete electrophoretic blot of representative bands of PPARγ level in adipose tissue of Wistar rats. Figure containing complete electrophoretic blot of representative bands of PPARγ level shown in Figure 2. In this file we indicate the experimental group related to each band. (PDF 161 KB) [file 12944_2014_1191_MOESM2_ESM.pdf]

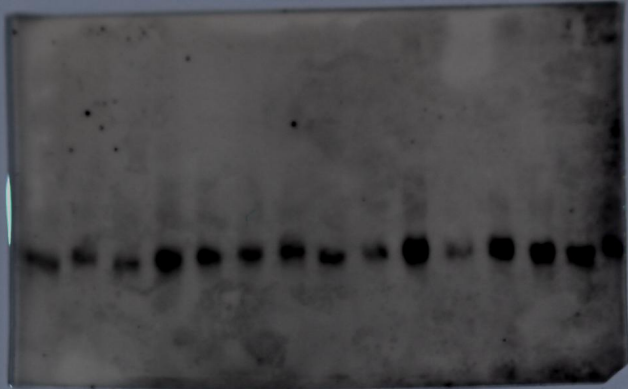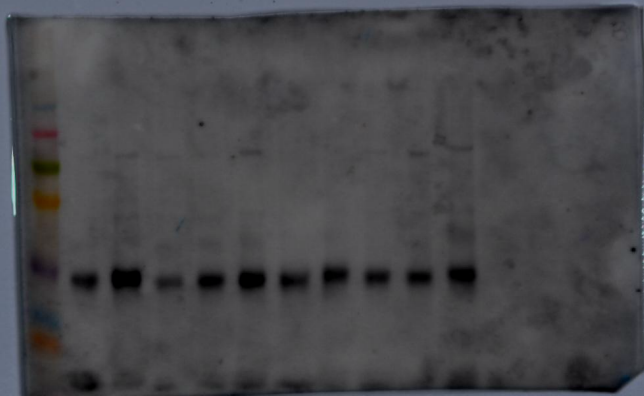

Supplement: Supplementary file 3 — Additional file 3:Complete electrophoretic blot of representative bands of β-tubulin (loading control) level in adipose tissue of Wistar rats. Figure containing complete electrophoretic blot of representative bands of β-tubulin level shown in Figure 2. (PDF 746 KB) [file 12944_2014_1191_MOESM3_ESM.pdf]

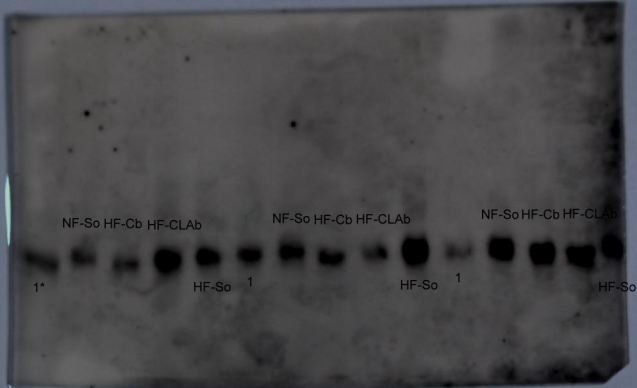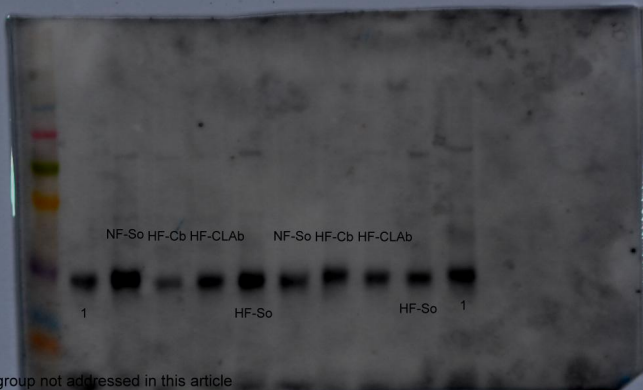

\* 1: Experimental group not addressed in this article

Supplement: Supplementary file 4 — Additional file 4:Complete electrophoretic blot of representative bands of β-tubulin level (loading control) in adipose tissue of Wistar rats. Figure containing complete electrophoretic blot of representative bands of β-tubulin level shown in Figure 2. In this file we indicate the experimental group related to each band. (PDF 80 KB) [file 12944_2014_1191_MOESM4_ESM.pdf]

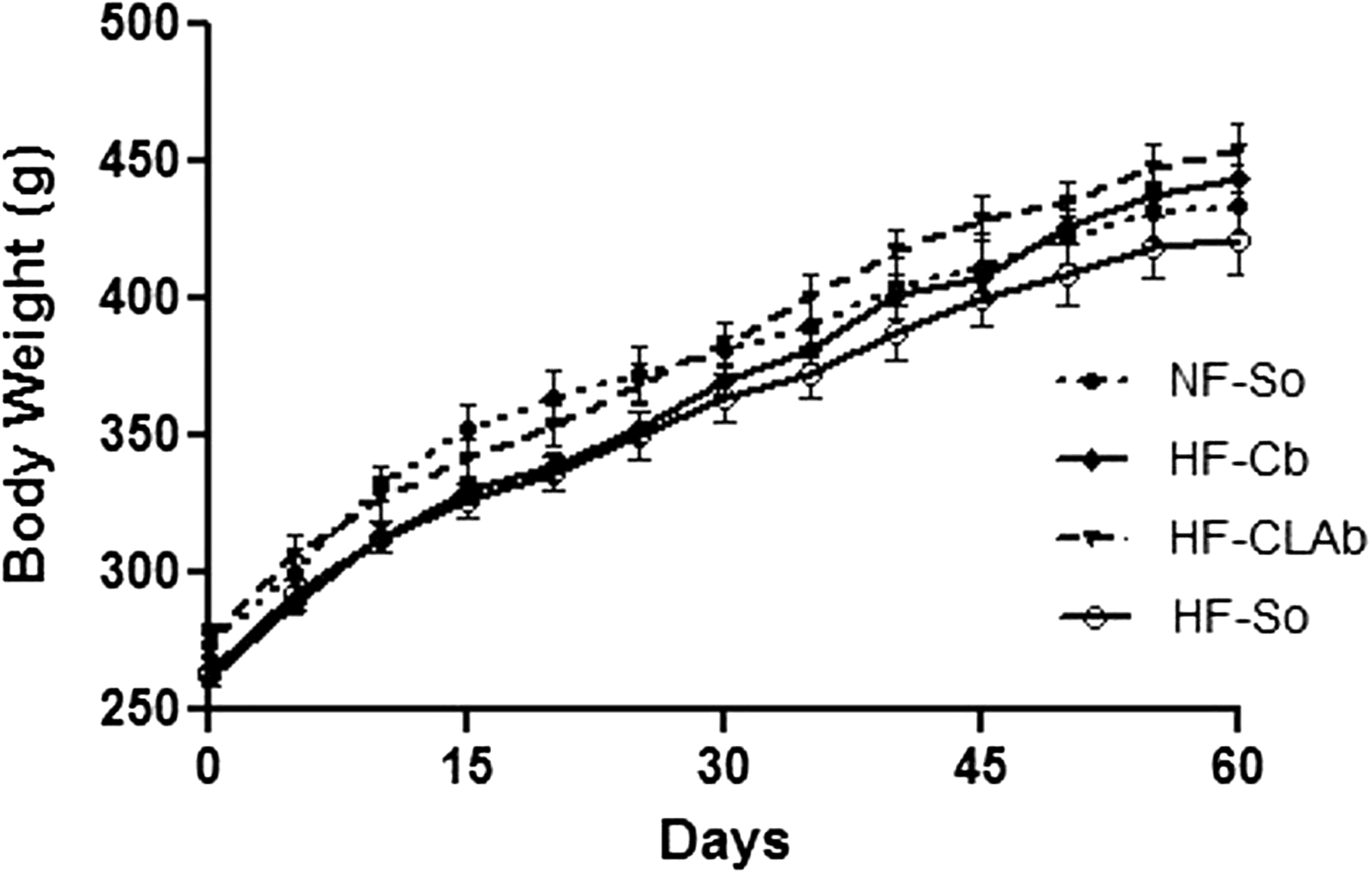

Supplement: Supplementary file 5 — Authors’ original file for figure 1 [file 12944_2014_1191_MOESM5_ESM.tif]

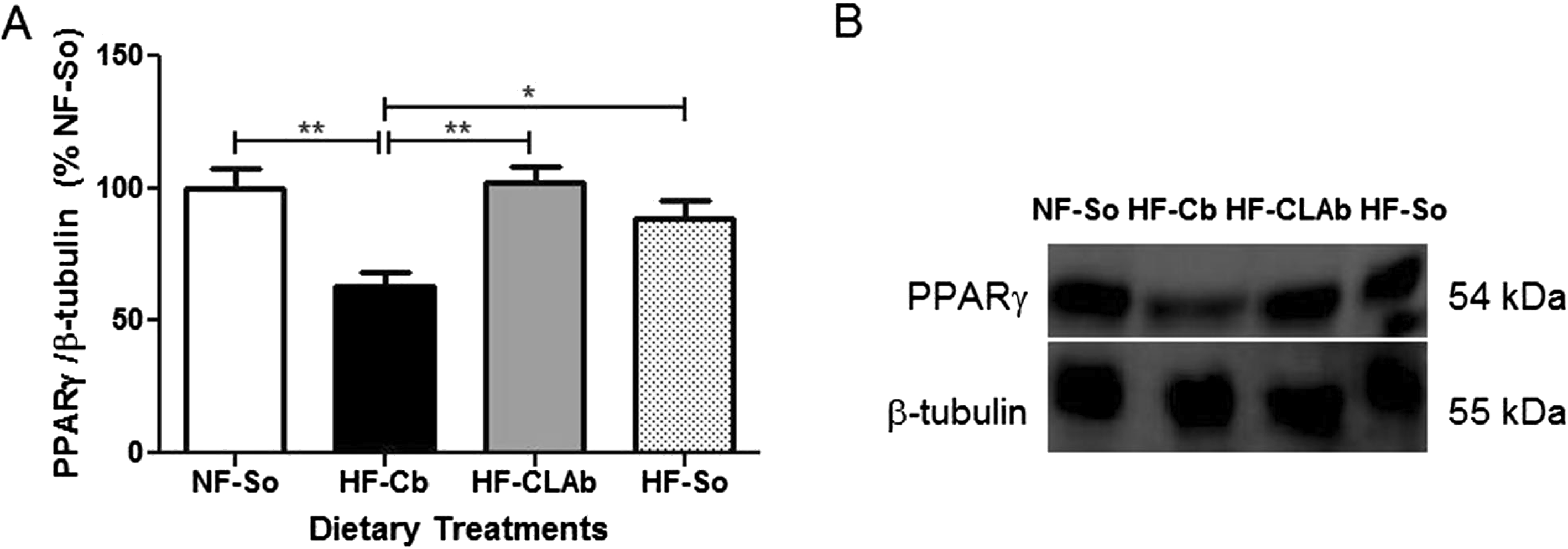

Supplement: Supplementary file 6 — Authors’ original file for figure 2 [file 12944_2014_1191_MOESM6_ESM.tif]

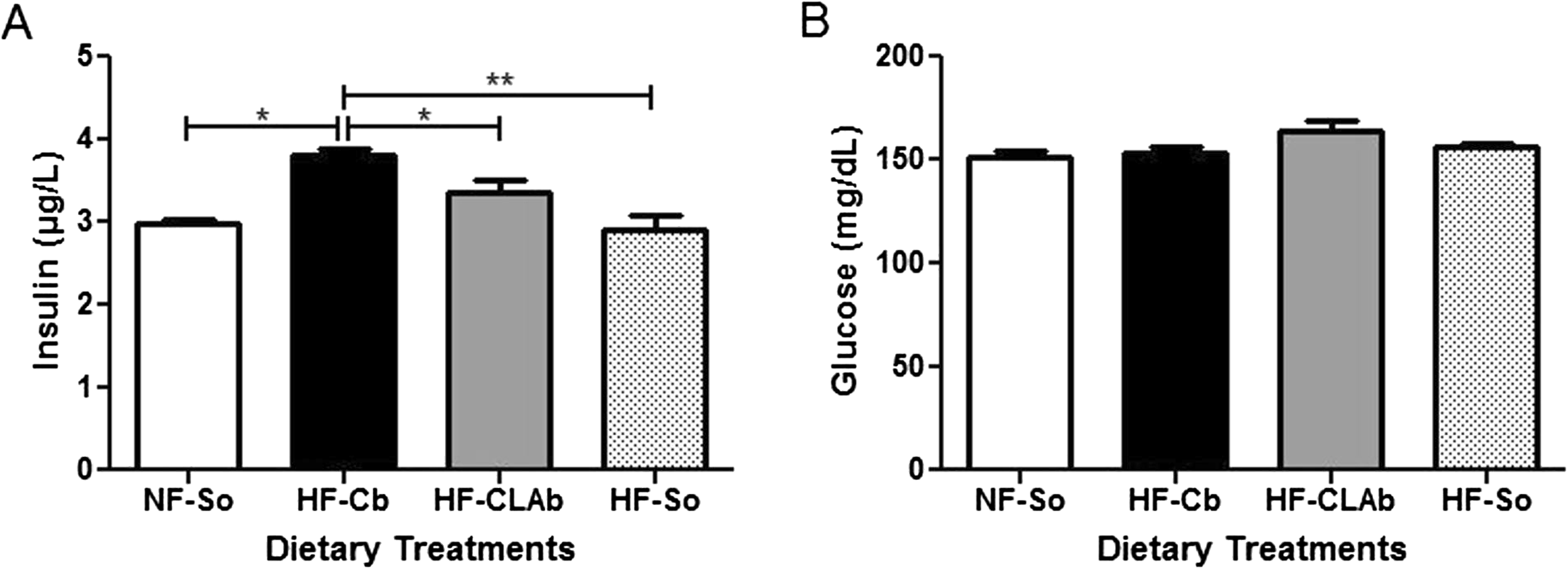

Supplement: Supplementary file 7 — Authors’ original file for figure 3 [file 12944_2014_1191_MOESM7_ESM.tif]

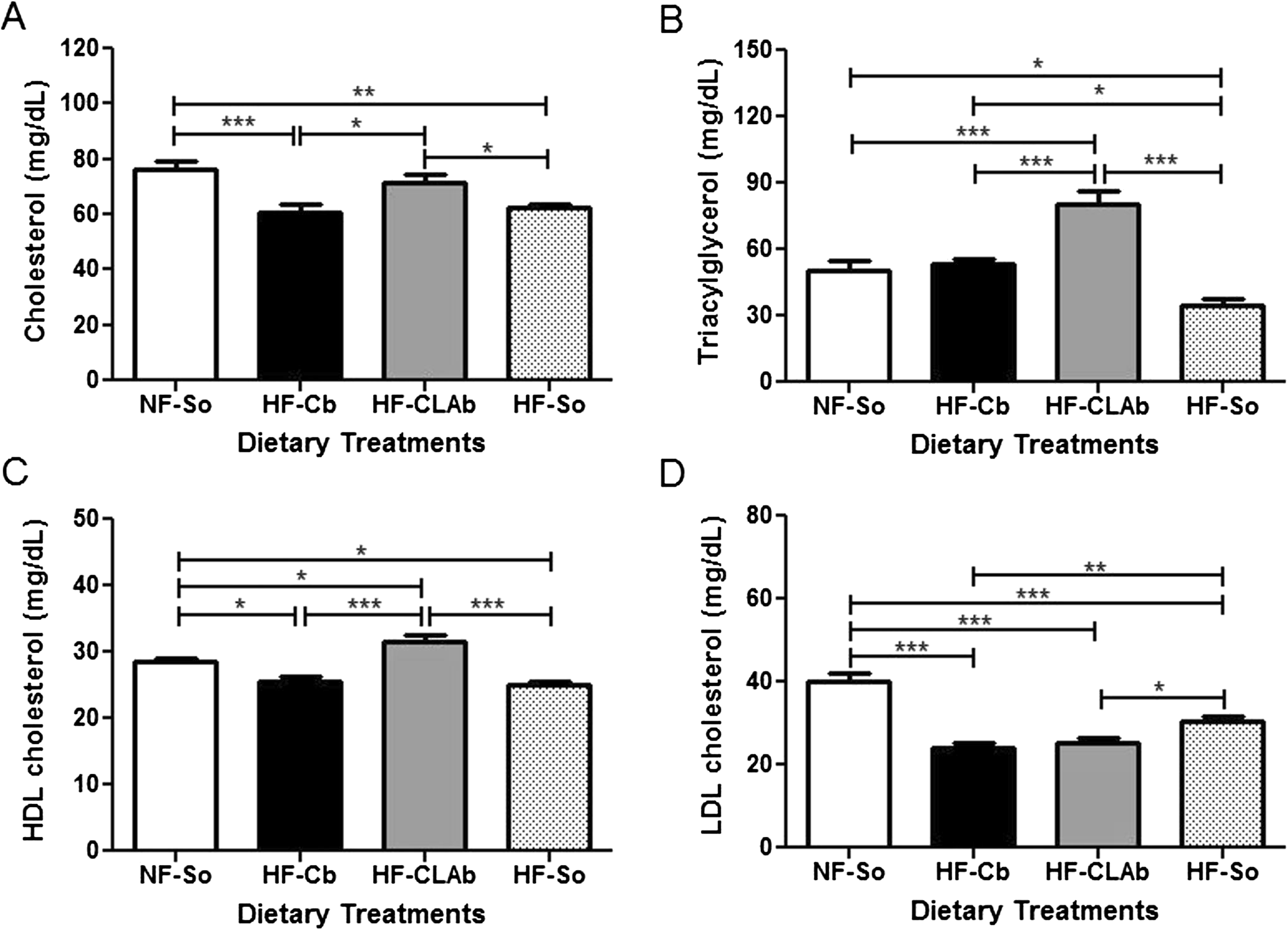

Supplement: Supplementary file 8 — Authors’ original file for figure 4 [file 12944_2014_1191_MOESM8_ESM.tif]
